# Housing Instability Following Medical Debt Exposure Among US Adults, 2023 to 2025

**DOI:** 10.1001/jamanetworkopen.2025.53617

**Published:** 2026-01-12

**Authors:** Kyle J. Moon, Sabriya L. Linton, Elizabeth A. Stuart, Sandro Galea, Catherine K. Ettman

**Affiliations:** 1 Department of Mental Health, Johns Hopkins Bloomberg School of Public Health, Baltimore, Maryland; 2 Department of Biostatistics, Johns Hopkins Bloomberg School of Public Health, Baltimore, Maryland; 3 Department of Health Policy and Management, Johns Hopkins Bloomberg School of Public Health, Baltimore, Maryland; 4 School of Public Health, Washington University in St Louis, St Louis, Missouri

## Abstract

**Importance:**

Medical debt may place patients at heightened risk of housing instability as a result of increased financial strain or extraordinary collection actions.

**Objective:**

To estimate the association of medical debt with housing instability in the subsequent year.

**Design, Setting, and Participants:**

Cohort study in the US from 2023 to 2025 including adults participating in the Cumulative Life Stressors Impact on Mental Health and Well Being Study, a nationally representative, longitudinal panel.

**Exposures:**

Past-year medical debt self-reported in 2024.

**Main Outcomes and Measures:**

Past-year housing instability (ie, difficulty paying rent or mortgage, eviction, or foreclosure) self-reported in 2025. Propensity score weighting was used to account for differences between adults with and without medical debt, using data from the year before medical debt (2023). Survey weights were used to account for complex survey design.

**Results:**

Among 1515 adults (mean [SD] age, 52.2 [16.2] years; 739 [49.7%] female; 138 [11.5%] Black, 203 [15.1%] Latine, and 1082 [65.0%] non-Hispanic White), 240 (16.4%) reported medical debt in 2024. The unadjusted prevalence of subsequent housing instability was markedly higher among adults with medical debt, compared with those without medical debt (23.5%; 95% CI, 18.2%-29.9% vs 5.8%; 95% CI, 3.6%-9.4%). Using doubly robust estimation to adjust for confounders, medical debt was associated with an increase of 7.0 (95% CI, 5.2-8.8) percentage points in the probability of housing instability in the subsequent year.

**Conclusions and Relevance:**

This cohort study found that any medical debt was associated with a significant increase in housing instability in the following year. Inquiry into efforts to prevent and mitigate the potential consequences of medical debt, including housing instability, are warranted.

## Key Points

**Question:**

Is medical debt associated with housing instability?

**Findings:**

This nationally representative cohort study of 1515 US adults estimated that any medical debt was associated with a 7–percentage point increase in the probability of experiencing housing instability in the subsequent year.

**Meaning:**

These findings suggest medical debt undermines assets and imperils future housing stability, which may, in turn, worsen health for adults carrying medical debt.

## Teaser

This cohort study evaluates the association between medical debt and housing instability in the subsequent year.

## Introduction

Over the past several decades, medical debt has become an increasingly common problem among US residents.^1,2,3^ An estimated 11% to 18% of US adults carry medical debt,^1,4,5^ with a cumulative burden estimated at $220 billion as of 2021.^6^ This problem emerges from a combination of high health care prices, uninsurance or underinsurance, and high levels of cost-sharing that leave patients bearing exorbitant out-of-pocket costs.^1,5^ Prior studies have documented an association between medical debt and delayed and forgone health care services,^4,7,8,9,10^ but medical debt may also exacerbate social drivers of health,^5^ with critical implications for housing, in particular. Housing has long been recognized as a critical asset for health promotion, with robust evidence of the relationship between housing and health at both the individual-level and population-level.^11,12,13,14^ Medical debt may affect housing instability as a result of extraordinary collection actions, changes in credit scores, or financial strain, potentially inducing and exacerbating cycles of poor health.

Beginning in the 1980s, some health systems have embraced aggressive tactics for debt collection, including but not limited to reporting debt to credit bureaus, suing patients, garnishing wages, placing liens on properties, and foreclosing on patients’ homes.^2,3,15,16,17^ All of these extraordinary collection actions can have severe consequences for patients’ current or future housing stability.^18,19^ For example, carrying medical debt may lead to lower credit scores,^20,21,22^ which, in turn, can limit access to rental properties and affordable mortgages.^23,24,25^ Prior studies have noted associations of medical debt with both foreclosures and homelessness. Among adults undergoing foreclosure, 28% to 41% of individuals cited medical debt as contributing to the foreclosure of their home,^26,27^ and among adults experiencing homelessness, nearly one-third believed medical debt to be, in part, responsible for their current situation.^28^ In addition to the impacts of extraordinary collection actions, medical debt may undermine access to assets,^29^ reduce finances available to cover essential needs,^30,31,32^ deplete savings,^30,31^and induce a cascade of consequences that hinders economic advancement.^19^

Important knowledge gaps in our understanding of medical debt and housing stability remain. Past studies have largely been among people experiencing housing instability (eg, homelessness or foreclosure),^19,26,27,28^ limiting inference about the consequences of medical debt in and of itself. In addition, most existing studies are limited to samples in select metropolitan areas that may not be generalizable to the entire US population.^19,26,27,28^ Most existing studies have used cross-sectional designs, and thus cannot account for the temporality of exposure and outcome.^19,28^ Finally, existing longitudinal studies have used a small set of baseline confounders that do not fully account for fundamental differences between adults with and without medical debt.^5^

Leveraging 3 years (2023-2025) of panel data from a nationally representative sample of US adults with detailed data on sociodemographics, assets, and housing, this study aims to estimate the association of medical debt with subsequent housing instability. Understanding the association between medical debt and housing instability has critical implications, particularly in an evolving policy environment marked by dramatic, ongoing changes to the coverage of health care and social services.^33,34,35,36^

## Methods

### Study Population and Setting

This cohort study uses data from a nationally representative sample of US adults who participated in waves 4 (2023), 5 (2024), and 6 (2025) of the Cumulative Life Stressors Impact on Mental Health and Well Being (CLIMB) Study. As described elsewhere,^37,38^ CLIMB participants are recruited from the standing AmeriSpeak panel, which uses a 2-stage probability sampling design. The AmeriSpeak panel uses an address-based sampling frame to yield a nationally representative sample, recruiting participants through mailings, telephone contacts, and in-person follow-up. Between March and April annually, individuals are invited to participate in the CLIMB study via email and/or telephone; participants provide informed consent before initiating the CLIMB survey. Responses are then weighted to align the sample with the US adult population using benchmarks from the US Current Population Survey for age, sex, race and ethnicity, Census geographic division, educational attainment, age by sex, age by race and ethnicity, and race and ethnicity by sex. The CLIMB study was deemed exempt by the institutional review board (IRB) at NORC at the University of Chicago, and secondary analysis of deidentified data was not considered to be human participants research by the IRB at Johns Hopkins Bloomberg School of Public Health. Reporting adheres to the Strengthening the Reporting of Observational Studies in Epidemiology (STROBE) reporting guideline.^39^

In 2023, 7802 adults were invited to participate, of whom 2479 (31.8%) responded. Of the 2479 participants in 2023, 2020 (81.5%) responded in 2024, with 1744 (70.4%) also responding in 2025. We restricted the sample to individuals who participated in all 3 waves and further restricted to those with no missing data for the exposure and outcome of interest. We conducted a complete case analysis, excluding participants with missing data on any covariates, yielding the final analytic sample. To assess the validity of the complete case approach, we compared the characteristics of participants included vs excluded (due to missing covariate data) in the analytic sample, finding the 2 groups to be largely comparable, although slight differences were observed in the distribution of educational attainment, household savings, sex, and metropolitan statistical area designation of residence (eTable 1 in Supplement 1).

### Measures

The primary exposure of interest was medical debt in 2024, defined as an endorsement of the survey item during wave 5, “In the past 12 months, did you have problems paying or an inability to pay any medical bills, such as bills for doctors, dentists, medication, or home care? Please include any bills you have had problems paying over the past 12 months, even if the initial bill was incurred more than 12 months ago,” consistent with other work.^8,40,41^ The primary outcome of interest was subsequent housing instability, using responses from 2025 (wave 6). Housing instability has been variably defined in the literature, but in studies using national survey data, it is typically defined by difficulty paying housing costs.^42,43,44^ To optimize statistical power, we constructed a composite variable that encompasses past-year eviction, foreclosure, difficulty paying rent, or loss of housing, meaning if an individual experienced any of these stressors, they would be coded as having experienced housing instability, consistent with extant literature.^44^

The following covariates were captured based on responses in 2023 (wave 4), before medical debt exposure: sex, age, racialized group, educational attainment, marital status, parental status, household size, employment status, health insurance, household income, household savings, housing tenure, prior housing instability, prior medical debt, geographic region, and urbanicity of residence. All aforementioned sociodemographic characteristics were measured by participant self-report. In the CLIMB survey, participants were asked to select all of the following categories they consider themselves to be: American Indian or Alaskan Native; Asian Indian; Black or African American; Chinese; Filipino; Japanese; Korean; other Asian, Native Hawaiian, Guamanian or Chamorro, Samoan, other Pacific Islander; Vietnamese; White, or some other race. To limit any cell size with less than 3%, categories were combined into the following mutually exclusive categories for the purposes of this analysis: Hispanic, non-Hispanic Black, non-Hispanic White, and other, where other includes those who identify as American Indian or Alaska Native, Asian (ie, Asian Indian, Chinese, Filipino, Japanese, Korean, Vietnamese, and other Asian), Native Hawaiian or Other Pacific Islander (ie, Guamanian or Chamorro and Samoan), multiracial, or other.

### Statistical Analysis

We first computed survey-weighted descriptive statistics of our total sample and stratified by medical debt in 2024, presenting unweighted frequencies and weighted percentages. Next, to account for differences between adults with and without medical debt and ensure exchangeability on observed factors, we used a propensity score approach. More specifically, we used weighting by the odds to estimate the average treatment effect on the treated, whereby adults with medical debt receive a weight of 1 and adults without medical debt are weighted by their odds of exposure (ie, their propensity score).^45,46^ Propensity scores were estimated using generalized boosted models (GBM), a machine learning method that uses a series of regression trees,^47^ with the dependent variable being a dichotomous measure of medical debt in 2024 and the preexposure covariates described previously as independent variables.

Finally, to estimate the association of medical debt with subsequent housing instability, we fit survey- and propensity score-weighted logistic regression models that adjust for all preexposure covariates included in the propensity score estimation, yielding a doubly robust effect estimate.^45,48,49^ We report results as average marginal effects (AMEs) and use SEs that account for the complex survey design of CLIMB.

We performed all statistical analyses using R statistical software version 4.4.2 (R Project for Statistical Computing), including the following packages: survey,^50^ cobalt,^51^ MatchIt,^52^ WeightIt,^53^ marginaleffects,^54^ gtsummary,^55^ and EValue.^56^ Statistical tests were 2-sided, with significance defined as *P* < .05.

We performed several robustness checks to assess sensitivity to the propensity score approach used. First, we checked for extreme weights (eFigure 1 in Supplement 1) and used graphical distributions of the propensity scores and standardized mean differences (SMDs) between adults with and without medical debt to assess balance after weighting (eFigure 2 in Supplement 1). We then performed 90% winsorization, whereby weights below the 5th percentile or above the 95th percentile were replaced with the 5th or 95th percentile, respectively, and refit the main model. Next, we tested the sensitivity of our results to various propensity score approaches, including weighting by the odds with logistic regression, inverse probability of treatment weighting (IPTW) with logistic regression and GBM, and 1:1 nearest neighbor without replacement.

We also calculated the E-value to quantify how much unobserved confounding would be required to explain away the estimated association between medical debt and housing instability.^57^ Finally, we conducted a falsification test, whereby we evaluated the association between medical debt and an outcome that we suspect would be entirely unassociated with the exposure (past 12-month chatbot use), as spurious associations can result from preexisting differences between exposed and unexposed groups. This approach used the same weighting by the odds doubly robust approach as the primary analysis.

## Results

Among 1515 adults (mean [SD] age, 52.2 [16.2] years; 739 [49.7%] female; 138 [11.5%] Black, 203 [15.1%] Hispanic, and 1082 [65.0%] non-Hispanic White), the majority of participants identified as homeowners (69.5%), with annual household income of $60 000 or higher (56.6%) and less than $55 000 in household savings (56.2%). Those with medical debt, compared with those without medical debt, more often reported current employment (63.4% vs 57.0%), no health insurance (8.0% vs 4.1%), annual household income less than $60 000 (61.3% vs 39.8%), less than $5000 in household savings (51.9% vs 22.4%), and children aged less than 18 years in the home (39.7% vs 25.0%). Complete sociodemographic characteristics, stratified by medical debt exposure in 2024, are presented in the Table.

**Table.** Characteristics of Participants in the Full Analytic Sample, With Characteristics Measured in 2023^a^

Characteristic	Participants, No. (%)			*P* value^b^
	Overall (N = 1515)	No medical debt in 2024 (n = 1275)	Medical debt in 2024 (n = 240)	
Sex				
Male	776 (50.3)	676 (51.4)	100 (44.8)	<.001
Female	739 (49.7)	599 (48.6)	140 (55.2)	
Age, y				
18-34	256 (23.0)	213 (22.6)	43 (25.0)	<.001
35-44	292 (16.6)	237 (15.9)	55 (20.0)	
45-54	238 (15.9)	173 (13.8)	65 (26.6)	
55-64	328 (19.3)	276 (19.7)	52 (17.3)	
≥65	401 (25.2)	376 (28.0)	25 (11.1)	
Race and ethnicity				
Black	138 (11.5)	112 (11.6)	26 (11.2)	.11
Hispanic	203 (15.1)	165 (14.4)	38 (19.0)	
Non-Hispanic White	1082 (65.0)	917 (65.3)	165 (63.4)	
Other^c^	92 (8.3)	81 (8.8)	11 (6.3)	
Marital status				
Married	877 (53.5)	751 (54.3)	126 (49.3)	.46
Never married	331 (27.6)	277 (27.5)	54 (28.2)	
Divorced, separated, or widowed	307 (18.9)	247 (18.2)	60 (22.5)	
Children in the home				
No	1104 (72.6)	955 (75.0)	149 (60.3)	<.001
Yes, ≥1	411 (27.4)	320 (25.0)	91 (39.7)	
Household size				
1 Person	273 (16.9)	246 (17.9)	27 (11.6)	.09
2 Persons	562 (35.6)	488 (36.8)	74 (29.7)	
3-4 Persons	469 (31.0)	376 (29.5)	93 (38.5)	
≥5 Persons	211 (16.5)	165 (15.8)	46 (20.2)	
Educational attainment				
Less than high school	45 (5.7)	34 (5.4)	11 (7.6)	<.001
High school or equivalent	245 (28.4)	196 (26.8)	49 (36.5)	
Some college	581 (26.6)	467 (25.7)	114 (31.0)	
Bachelor’s degree	356 (21.9)	311 (22.8)	45 (17.3)	
Postgraduate degree	288 (17.4)	267 (19.3)	21 (7.7)	
Employment status				
Employed	903 (58.1)	749 (57.0)	154 (63.4)	<.001
Unemployed	131 (11.7)	106 (11.9)	25 (10.4)	
Retired	358 (21.7)	335 (24.1)	23 (9.5)	
Other	123 (8.5)	85 (6.9)	38 (16.7)	
Health insurance				
Commercial	853 (53.7)	722 (54.1)	131 (51.4)	<.001
Medicare	416 (26.9)	362 (27.5)	54 (24.1)	
Medicaid	120 (10.5)	94 (10.0)	26 (13.4)	
Uninsured	64 (4.7)	45 (4.1)	19 (8.0)	
Other	62 (4.1)	52 (4.3)	10 (3.2)	
Annual household income, $				
<30 000	224 (17.4)	177 (16.6)	47 (21.4)	<.001
30 000-59 999	385 (26.0)	292 (23.2)	93 (39.9)	
60 000-99 999	425 (26.6)	357 (26.8)	68 (26.1)	
≥100 000	481 (30.0)	449 (33.4)	32 (12.6)	
Household savings, $				
<5000	372 (27.3)	246 (22.4)	126 (51.9)	<.001
5000-19 999	233 (16.8)	186 (16.1)	47 (20.2)	
20 000-54 999	186 (12.1)	158 (11.7)	28 (14.1)	
55 000-99 999	127 (8.2)	115 (9.1)	12 (4.0)	
≥100 000	597 (35.6)	570 (40.7)	27 (9.8)	
Census region				
Northeast	223 (18.2)	185 (18.1)	38 (18.8)	.14
Midwest	414 (20.0)	346 (20.0)	68 (20.0)	
South	502 (38.5)	411 (37.5)	91 (44.1)	
West	376 (23.2)	333 (24.4)	43 (17.1)	
MSA designation				
Nonmetropolitan area	229 (14.6)	182 (13.6)	47 (19.7)	<.001
Metropolitan area	1286 (85.4)	1093 (86.4)	193 (80.3)	
Housing tenure				
Own	1072 (69.5)	936 (72.0)	136 (56.9)	<.001
Rent or other^d^	443 (30.5)	339 (28.0)	104 (43.1)	
Prior medical debt				
No	1280 (83.8)	1184 (92.8)	96 (37.8)	<.001
Yes	235 (16.2)	91 (7.2)	144 (62.2)	
Prior housing instability				
No	1396 (90.6)	1222 (94.3)	174 (71.7)	<.001
Yes	119 (9.4)	53 (5.7)	66 (28.3)	
Housing instability (in 2025)				
No	1405 (91.3)	1225 (94.2)	180 (76.5)	<.001
Yes	110 (8.7)	50 (5.8)	60 (23.5)	

Abbreviation: MSA, metropolitan statistical area.

^a^ Presented are unweighted frequencies and survey-weighted percentages. These comparisons do not use the propensity score weights.

^b^ *P* values are reported for χ^2^ tests with Rao-Scott second-order correction, comparing the sample proportions of covariates between adults with and without medical debt in 2024.

^c^ Other race and ethnicity includes those who identify as American Indian or Alaska Native, Asian, Native Hawaiian or Other Pacific Islander, multiracial, or other.

^d^ Other housing tenure refers to those who reported occupying their home without payment of cash rent.

A total of 240 individuals (16.4%; 95% CI, 15.1%-17.7%) reported carrying medical debt in 2024, and housing instability in 2025 was reported by 110 individuals (8.7%; 95% CI, 6.0%-12.5%), of whom 103 (7.8%; 95% CI, 6.1%-10.0%) reported rent burden and 15 (1.5%; 95% CI, 0.7%-3.0%) reported losing housing due to eviction or foreclosure; these were not mutually exclusive outcomes. In the total sample, 119 individuals reported prior experience of housing instability in 2023, of whom more than 2 in 5 (52 individuals) reported housing instability again in 2025.

**Figure 1.**
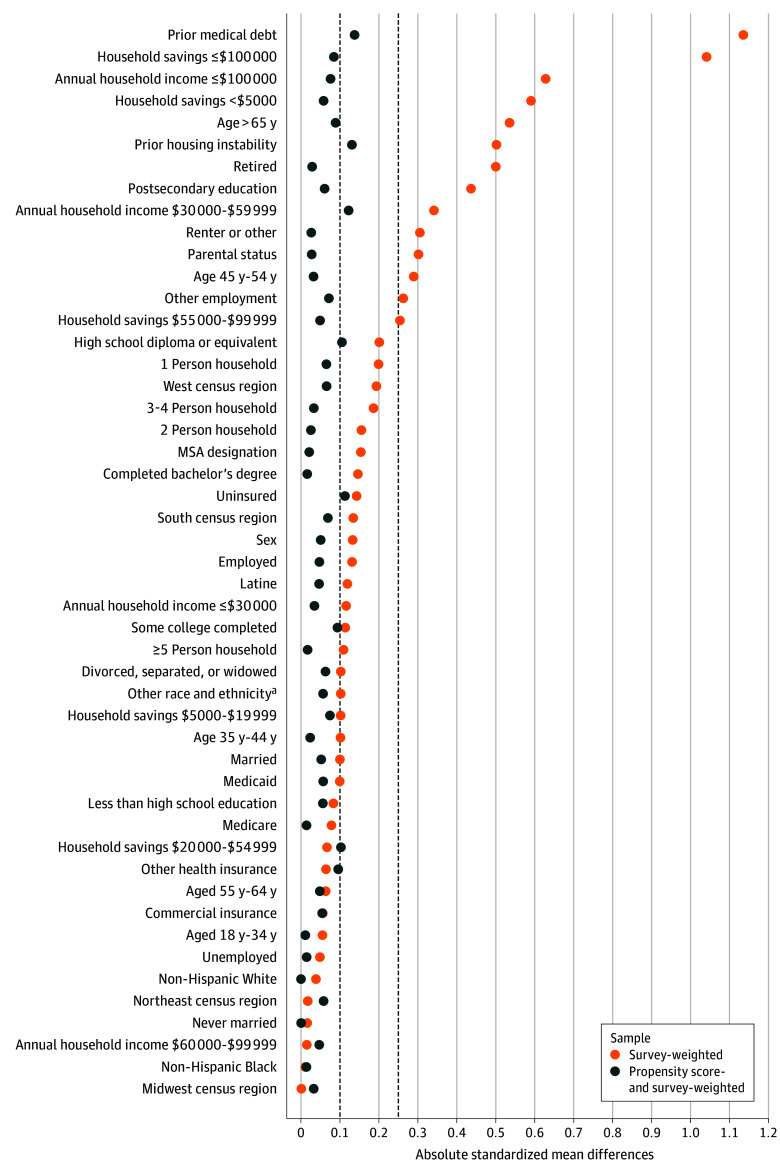
Premedical Debt Exposure Covariate Balance Before and After Propensity Score Weighting Absolute values of the standardized mean differences were calculated for all covariates to assess balance between adults with and without medical debt before and after applying propensity score weighting by the odds to estimate the average treatment effect on the treated. Propensity scores were estimated using generalized boosted models. Data are drawn from the Cumulative Life Stressors Impact on Mental Health and Well Being Study, a nationally representative panel of US adults. MSA indicates metropolitan statistical area. ^a^Other race and ethnicity includes those who identify as American Indian or Alaska Native, Asian, Native Hawaiian or Other Pacific Islander, multiracial, or other.

**Figure 2.**
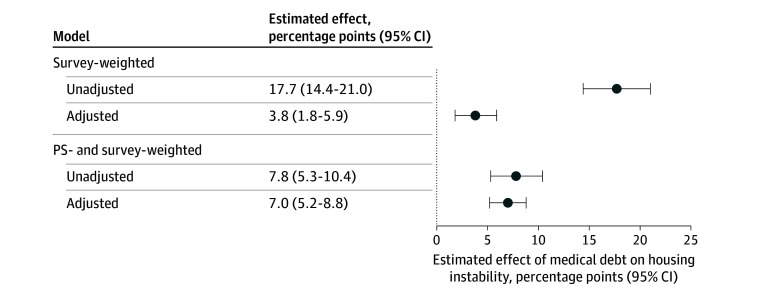
Probability of Housing Instability Following Medical Debt Exposure Compared With Adults Without Medical Debt Average marginal effects with 95% CIs obtained from logistic regression are presented to the left of the plot. Data are drawn from the Cumulative Life Stressors Impact on Mental Health and Well Being Study, a nationally representative panel of US adults. PS indicates propensity score.

There were notable differences between adults with and without medical debt, particularly in prior experiences of housing instability, prior medical debt, and financial assets. Before propensity score weighting, the prevalence of housing instability was markedly higher among adults with medical debt, with 60 participants (23.5%; 95% CI 18.2%-29.9%) reporting housing instability in the subsequent year, compared with 50 (5.8%; 95% CI, 3.6%-9.4%) without medical debt. Propensity score weighting yielded acceptable balance in preexposure covariates between adults with and without medical debt (Figure 1), with absolute SMDs less than 0.25 for all covariates,^58^ including prior housing instability and prior medical debt. Using doubly robust estimation to adjust for observed confounders, incurring medical debt was associated with an increase of 7.0 (95% CI, 5.2-8.8) percentage points in the probability of subsequent housing instability (Figure 2). Full model estimates are available in eTable 2 in Supplement 1.

### Sensitivity Analyses

Using 90% winsorization and doubly robust estimation, we estimated that medical debt was associated with an increase of 6.5 (95% CI, 4.0-8.9) percentage points in the probability of subsequent housing instability, which is consistent with our main analysis; see eTable 3 in Supplement 1 for full model estimates. When comparing the various propensity score approaches (eFigure 3 in Supplement 1), weighting by the odds with GBM achieved the best balance. Other approaches demonstrated fair but not great balance, as SMD values were greater than 0.25 for several of the covariates.^58^ However, when refitting the main model with each of the alternative methods, estimates were stable across all 4 propensity score approaches (eTable 4 in Supplement 1). Full model estimates from each of the alternative propensity score approaches are shown in eTables 5 through 8 in Supplement 1, and characteristics of the matched cohort are shown in eTable 9 in Supplement 1.

Next, to assess sensitivity to unmeasured confounding, we estimated an e-value of 3.3. This implies that an unmeasured confounder would need to be associated with both medical debt and housing instability by at least 3.3-fold to explain away the effect of medical debt on subsequent housing instability.^57^ Finally, we performed a placebo test, finding no association between medical debt and subsequent chatbot use (AME, 2.4; 95% CI, −1.8 to 6.6). Full model estimates are shown in eTable 10 in Supplement 1.

## Discussion

In this nationally representative cohort of US adults surveyed from 2023 to 2025, incurring medical debt was associated with a 5 to 9 percentage point increase in the probability of experiencing housing instability in the subsequent year. While the high costs of care in the US may discourage persons from seeking health services altogether,^4,7,8^ persons who do access care can experience financial hardship, including medical debt.^5^ These findings highlight the toll of medical debt, which undermines assets and may compromise future housing stability, underscoring the urgent and sustained need for policy interventions to address medical debt.

Several studies have highlighted how medical debt contributes to financial strain, evictions, foreclosures, and homelessness.^5,19,26,27,28^ We extend upon previous work, using longitudinal data from a nationally representative sample to estimate the association of medical debt with subsequent experiences of housing instability with the most recent data available (2023-2025). In terms of the prevalence of housing instability, our estimate of 8.7% is lower than what has been reported by others (12%-17%).^43,44^ For the prevalence of medical debt, we estimated 16.4% of adults carried medical debt in 2024, which is consistent with prior studies that have reported prevalence estimates of 11% to 18%.^1,4,5^ Compared with those without medical debt, adults carrying medical debt had larger households, often including children, which may contribute to the high prevalence of unstable housing experienced by children and families.^44^ Emerging evidence suggests that government housing assistance may blunt the impacts of medical financial hardship, but further work is needed to inform policy interventions that protect against medical debt and promote housing stability.^59^

These findings have critical implications, especially in an evolving policy environment. For one, the 2025 budget reconciliation act, signed into law on July 4, 2025, represents a considerable rollback of health insurance coverage in US history, marked by significant changes to the Medicaid program, including work requirements and eligibility redeterminations every 6 months, among other reforms.^33^ As a result, an estimated 7.6 million individuals in the US are projected to lose health insurance coverage by 2034.^62^ These changes raise concerns about the affordability of care^33^ that ultimately may contribute to the growing burden of medical debt, potentially igniting a cascade of consequences that imperil housing stability. Additionally, in July 2025, a federal judge reversed a final rule by the US Consumer Financial Protections Bureau that would have removed medical debt from credit reports. The projections associated with this final rule have been mixed, with some reporting no effect on credit outcomes,^20^ while others anticipated an average change of 20 points to consumers’ credit scores that would have expanded access to affordable mortgages.^21^ Moreover, these initiatives would not have affected health systems’ ability to engage in extraordinary collection actions, such as reporting debt to credit bureaus, suing patients, garnishing wages, placing liens on properties, and foreclosing on patients’ homes.^2,3,15^ In the absence of change, there remains an urgent need for policies and programs to address medical debt and its collateral consequences, namely housing instability. Many state-level policies are under way,^63,64^ and evaluations of these efforts should consider the impacts on patients’ housing security.

### Limitations

This study is subject to several limitations. First, both the exposure and outcome were collected by participant self-report and may suffer from recall bias. Second, there is a potential selection bias, as individuals were required to have responded to the CLIMB survey in all 3 years to be included and differential dropout over time could bias estimates. For example, if those with the most severe housing instability are more likely to be lost to follow-up, this would result in an underestimate of the true association. Third, due to limited statistical power, we could not stratify the outcome to assess differences by type of housing instability (eg, foreclosure, eviction, or rent burden). Relatedly, the number of foreclosures and evictions in this sample may have been undercounted, as judicial proceedings are multistep processes that may extend beyond 1 year, and survey items that ask about eviction may not capture the full extent of forced moves or informal evictions^60,61^; this may underestimate the association between medical debt and housing instability. Fourth, with limited statistical power, we aggregated multiple racial and ethnic groups into an other category, which is difficult to interpret and hindered our ability to assess effect measure modification by racialized group, as well as geographic region, employment status, and housing tenure. Future research is needed to better understand heterogenous effects of medical debt to identify those that would most benefit from programs to address medical debt.

## Conclusions

In this nationally representative cohort study of US adults, medical debt heightened the risk of future housing instability. Given the foundational importance of housing for individual and population health,^11,12,13,14^ this may contribute to the poor health associated with medical debt,^65^ which is an increasingly common problem patients face in paying for health care.^1,10^ Policy interventions are needed to address the escalating problem of medical debt and its consequences, potentially including housing instability.

## Notes

Supplement 1. eFigure 1. Propensity Score Weight Distribution of Adults Without Medical Debt eFigure 2. Distribution of Propensity Scores by Medical Debt Exposure eFigure 3. Love Plot Comparing Covariate Balance After Various Propensity Score Estimation Approaches eTable 1. Participant Characteristics, by Missing Covariate Data Status eTable 2. Full Regression Output From Main Model Specification eTable 3. Full Regression Output From Model With 90% Winsorization eTable 4. Association of Medical Debt With Subsequent Housing Instability, Using Propensity Score- and Survey-Weighted Logistic Regression Models eTable 5. Full Regression Output From Model Using Propensity Score Weighting by the Odds With Logistic Regression to Estimate ATT eTable 6. Full Regression Output From Model Using Inverse Probability of Treatment Weighting With Generalized Boosted Models to Estimate ATE eTable 7. Full Regression Output From Model Using Inverse Probability of Treatment Weighting With Logistic Regression to Estimate ATE eTable 8. Full Regression Output From Model Using 1:1 Nearest Neighbor Propensity Score Matching Without Replacement eTable 9. Matched Cohort of Individuals With and Without Medical Debt, Using 1:1 Nearest Neighbor Propensity Score Matching eTable 10. Average Marginal Effects From Covariates in the Falsification Test, Using ChatGPT Use in 2025 as the Placebo Outcome

Supplement 2. Data Sharing Statement
