# Supplementary material for: Housing Instability Following Medical Debt Exposure Among US Adults, 2023 to 2025
Source: JAMA Netw Open. 2026 Jan 12;9(1):e2553617. doi: 10.1001/jamanetworkopen.2025.53617 (PMC12797096; doi:10.1001/jamanetworkopen.2025.53617)
Supplement: Supplement 1. — eFigure 1. Propensity Score Weight Distribution of Adults Without Medical Debt eFigure 2. Distribution of Propensity Scores by Medical Debt Exposure eFigure 3. Love Plot Comparing Covariate Balance After Various Propensity Score Estimation Approaches eTable 1. Participant Characteristics, by Missing Covariate Data Status eTable 2. Full Regression Output From Main Model Specification eTable 3. Full Regression Output From Model With 90% Winsorization eTable 4. Association of Medical Debt With Subsequent Housing Instability, Using Propensity Score- and Survey-Weighted Logistic Regression Models eTable 5. Full Regression Output From Model Using Propensity Score Weighting by the Odds With Logistic Regression to Estimate ATT eTable 6. Full Regression Output From Model Using Inverse Probability of Treatment Weighting With Generalized Boosted Models to Estimate ATE eTable 7. Full Regression Output From Model Using Inverse Probability of Treatment Weighting With Logistic Regression to Estimate ATE eTable 8. Full Regression Output From Model Using 1:1 Nearest Neighbor Propensity Score Matching Without Replacement eTable 9. Matched Cohort of Individuals With and Without Medical Debt, Using 1:1 Nearest Neighbor Propensity Score Matching eTable 10. Average Marginal Effects From Covariates in the Falsification Test, Using ChatGPT Use in 2025 as the Placebo Outcome [file jamanetwopen-e2553617-s001.pdf]

Supplemental Online Content

Moon KJ, Linton SL, Stuart EA, Galea S, Ettman CK. Housing instability following medical debt exposure among US adults, 2023 to 2025. *JAMA Netw Open*. 2026;9(1):e2553617. doi:10.1001/jamanetworkopen.2025.53617

eFigure 1. Propensity Score Weight Distribution of Adults Without Medical Debt

eFigure 2. Distribution of Propensity Scores by Medical Debt Exposure

eFigure 3. Love Plot Comparing Covariate Balance After Various Propensity Score Estimation Approaches

eTable 1. Participant Characteristics, by Missing Covariate Data Status

eTable 2. Full Regression Output From Main Model Specification

eTable 3. Full Regression Output From Model With 90% Winsorization

eTable 4. Association of Medical Debt With Subsequent Housing Instability, Using Propensity Score- and Survey-Weighted Logistic Regression Models

eTable 5. Full Regression Output From Model Using Propensity Score Weighting by the Odds With Logistic Regression to Estimate ATT

eTable 6. Full Regression Output From Model Using Inverse Probability of Treatment Weighting With Generalized Boosted Models to Estimate ATE

eTable 7. Full Regression Output From Model Using Inverse Probability of Treatment Weighting With Logistic Regression to Estimate ATE

eTable 8. Full Regression Output From Model Using 1:1 Nearest Neighbor Propensity Score Matching Without Replacement

eTable 9. Matched Cohort of Individuals With and Without Medical Debt, Using 1:1 Nearest Neighbor Propensity Score Matching

eTable 10. Average Marginal Effects From Covariates in the Falsification Test, Using ChatGPT Use in 2025 as the Placebo Outcome

This supplemental material has been provided by the authors to give readers additional information about their work.

© 2026 Moon KJ et al. *JAMA Network Open*.

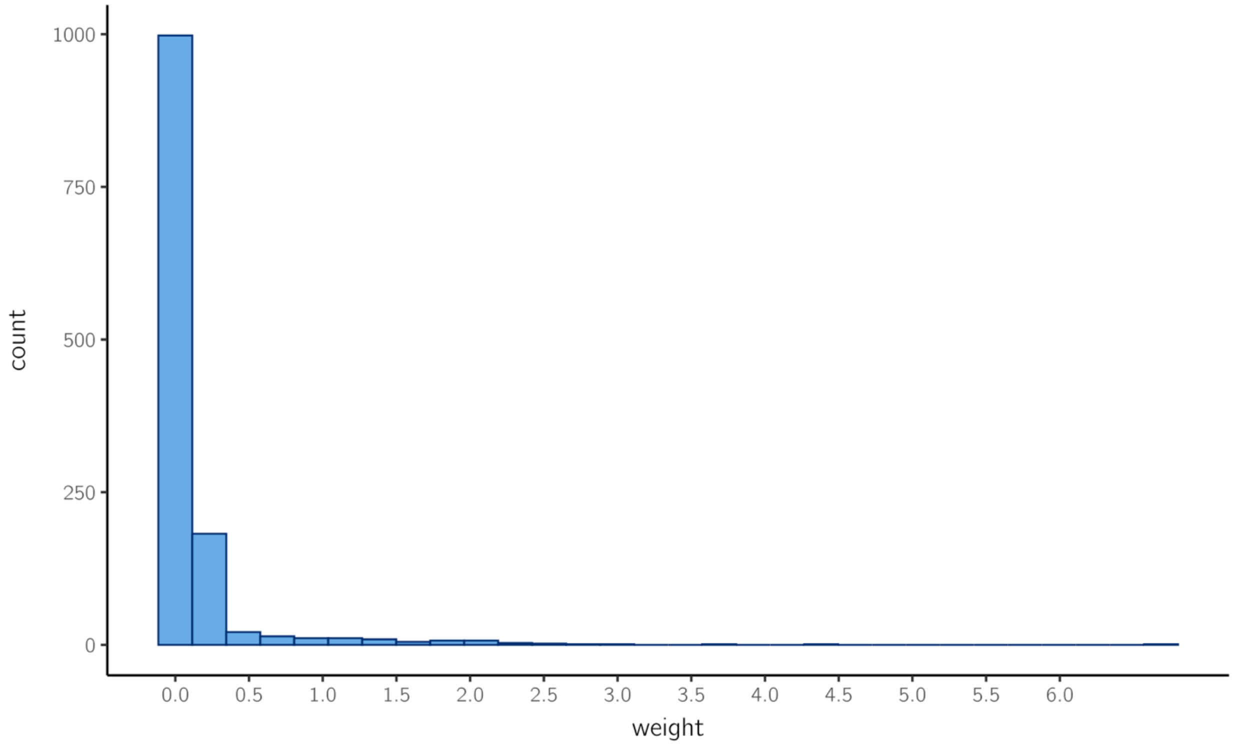

eFigure 1. Propensity Score Weight Distribution of Adults Without Medical Debt
This histogram shows propensity score weights, estimated with generalized boosted models, for adults without medical debt in 2024 (n = 1275). All adults that reported carrying medical debt in 2024 (n = 240) were each given a weight equal to one. Data are drawn from the CLIMB Study, a nationally representative panel of U.S. adults.

© 2026 Moon KJ et al. *JAMA Network Open*.

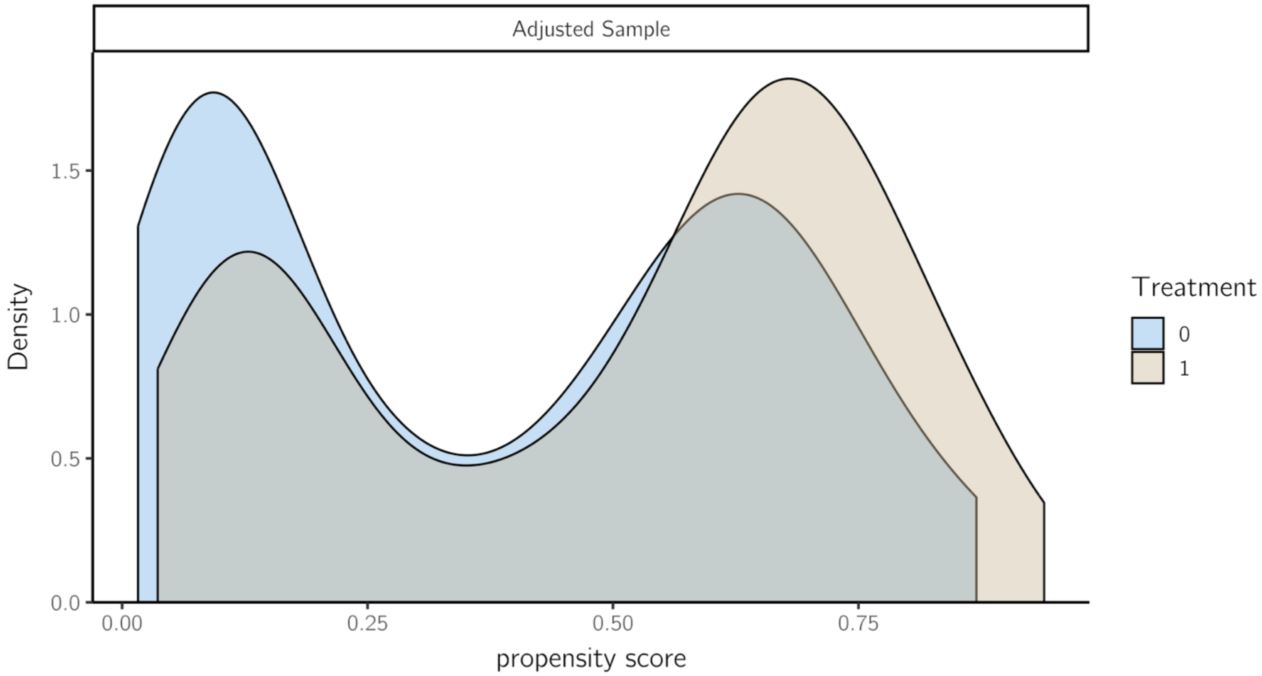

eFigure 2. Distribution of Propensity Scores by Medical Debt Exposure

Shown is the distribution of propensity scores between adults with and without medical debt exposure after using propensity score weighting by the odds, with propensity scores estimated using generalized boosted models. “Treatment” in this context refers to medical debt exposure, with adults without medical debt shown in blue and adults with medical debt in tan. Data are drawn from the CLIMB Study, a nationally representative panel of U.S. adults.

© 2026 Moon KJ et al. *JAMA Network Open*.

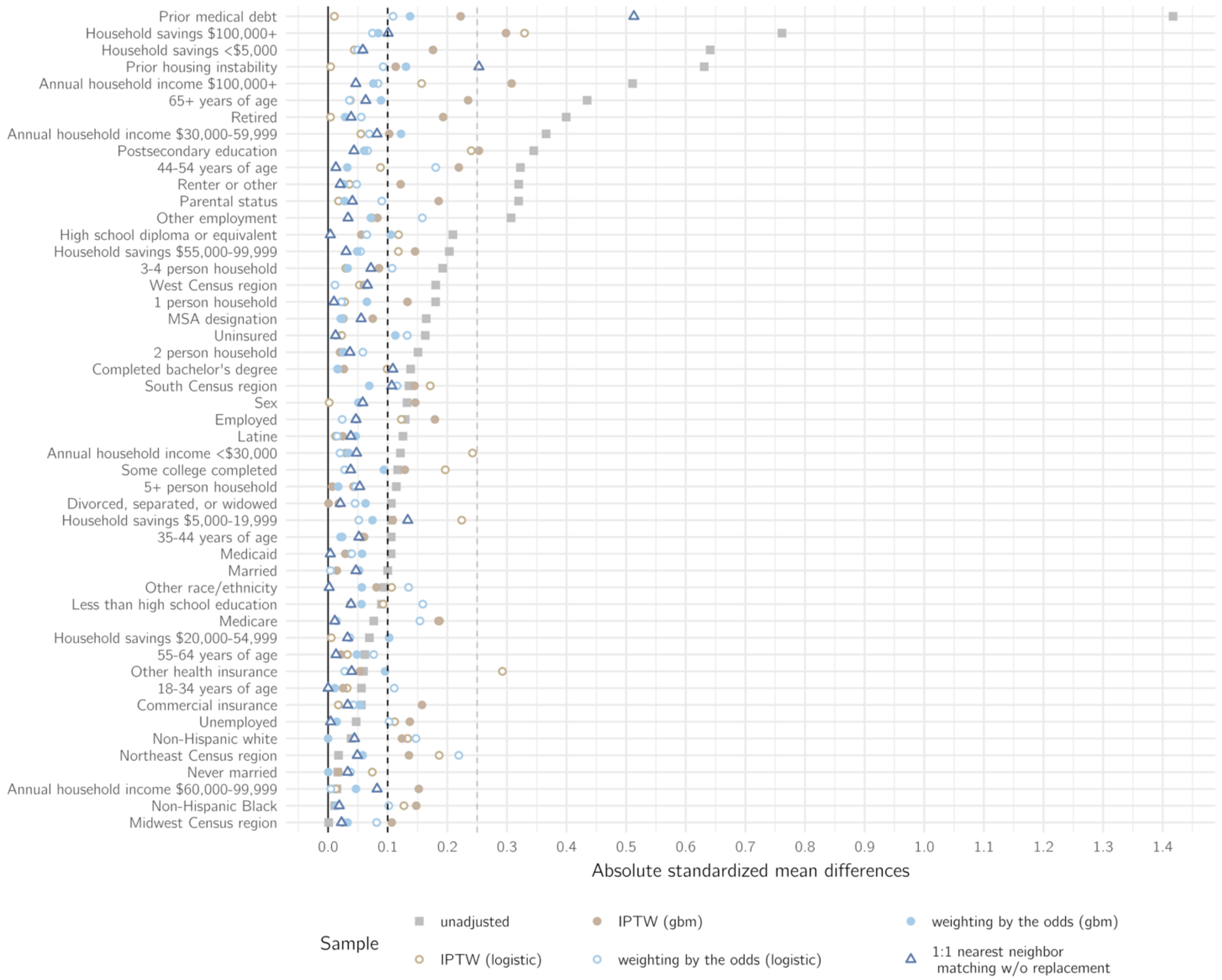

eFigure 3. Love Plot Comparing Covariate Balance After Various Propensity Score Estimation Approaches

Absolute standardized mean differences in pre-exposure covariates, measured in 2023, between adults with and without medical debt exposure are shown. Five different approaches were assessed to estimate and use propensity scores: (a) inverse probability of treatment weighting (IPTW) with logistic regression models, denoted by open circles in tan, (b) IPTW with generalized boosted models (GBM), denoted by filled circles in tan, (c) weighting by the odds with logistic regression models, denoted by open circles in light blue, (d) weighting by the odds with GBM, denoted by filled circles in light blue, and (e) 1:1 nearest neighbor matching without replacement with logistic regression, denoted by open triangles. Absolute standardized mean differences prior to propensity score adjustment are denoted by filled gray squares. Data are drawn from the CLIMB Study, a nationally representative panel of U.S. adults.

© 2026 Moon KJ et al. *JAMA Network Open*.

eTable 1. Participant Characteristics, by Missing Covariate Data Status [a]

	Overall (n = 1,633)	Analytic Sample [b] (n = 1,515)	Excluded due to missing variables [c] (n = 118)
Sex			
Male	823 [50%]	776 [51%]	47 [40%]
Female	810 [50%]	739 [49%]	71 [60%]
Age			
18–34 years	278 [17%]	256 [17%]	22 [19%]
35–44 years	313 [19%]	292 [19%]	21 [18%]
45–54 years	251 [15%]	238 [16%]	13 [11%]
55–64 years	356 [22%]	328 [22%]	28 [24%]
≥ 65 years	435 [27%]	401 [26%]	34 [29%]
Racialized group			
Black	150 [9%]	138 [9%]	12 [10%]
Latine	225 [14%]	203 [13%]	22 [19%]
Non-Hispanic white	1,158 [71%]	1,082 [71%]	76 [64%]
Other [d]	100 [6%]	92 [6%]	8 [7%]
Marital status			
Divorced, separated, or widowed	331 [20%]	307 [20%]	24 [20%]
Married	944 [58%]	877 [58%]	67 [57%]
Never married	358 [22%]	331 [22%]	27 [23%]
Children in the home			
No	1,193 [73%]	1,104 [73%]	89 [78%]
Yes, ≥ 1	436 [27%]	411 [27%]	25 [22%]
Missing	4	0	4
Household size			
1 person	294 [18%]	273 [18%]	21 [18%]
2 persons	597 [37%]	562 [37%]	35 [30%]
3–4 persons	508 [31%]	469 [31%]	39 [33%]
≥ 5 persons	234 [14%]	211 [14%]	23 [19%]
Educational attainment			
Less than high school	51 [3%]	45 [3%]	6 [5%]
High school	270 [17%]	245 [16%]	25 [21%]
Some college	636 [39%]	581 [38%]	55 [47%]
Bachelor's degree	366 [22%]	356 [23%]	10 [8%]
Postgraduate degree	310 [19%]	288 [19%]	22 [19%]
Employment status			
Employed	954 [59%]	903 [60%]	51 [46%]
Unemployed	144 [9%]	131 [9%]	13 [12%]
Retired	395 [24%]	358 [24%]	37 [33%]
Other	133 [8%]	123 [8%]	10 [9%]
Missing	7	0	7
Health insurance			
Commercial	902 [56%]	853 [56%]	49 [45%]
Medicaid	129 [8%]	120 [8%]	9 [8%]
Medicare	454 [28%]	416 [27%]	38 [35%]
Uninsured	70 [4%]	64 [4%]	6 [6%]
Other	68 [4%]	62 [4%]	6 [6%]
Missing	10	0	10
Annual household income			
< $30,000	256 [16%]	224 [15%]	32 [27%]
$30,000–59,999	416 [25%]	385 [25%]	31 [26%]
$60,000–99,999	460 [28%]	425 [28%]	35 [30%]

© 2026 Moon KJ et al. *JAMA Network Open*.

	Overall (n = 1,633)	Analytic Sample [b] (n = 1,515)	Excluded due to missing variables [c] (n = 118)
≥ $100,000	501 [31%]	481 [32%]	20 [17%]
Household savings			
< $5,000	397 [25%]	372 [25%]	25 [45%]
$5,000–19,999	244 [16%]	233 [15%]	11 [20%]
$20,000–54,999	194 [12%]	186 [12%]	8 [15%]
$55,000–99,999	132 [8%]	127 [8%]	5 [9%]
≥ $100,000	603 [38%]	597 [39%]	6 [11%]
Missing	63	0	63
Census region			
Northeast	241 [15%]	223 [15%]	18 [15%]
Midwest	449 [27%]	414 [27%]	35 [30%]
South	543 [33%]	502 [33%]	41 [35%]
West	400 [24%]	376 [25%]	24 [20%]
MSA designation [e]			
Nonmetropolitan area	256 [16%]	229 [15%]	27 [23%]
Metropolitan area	1,377 [84%]	1,286 [85%]	91 [77%]
Housing tenure			
Own	1,163 [71%]	1,072 [71%]	91 [77%]
Rent or other [f]	470 [29%]	443 [29%]	27 [23%]
Prior medical debt			
No	1,350 [85%]	1,280 [84%]	70 [92%]
Yes	241 [15%]	235 [16%]	6 [8%]
Missing	42	0	42
Prior housing instability			
No	1,507 [92%]	1,396 [92%]	111 [94%]
Yes	126 [8%]	119 [8%]	7 [6%]
Medical debt in 2024			
No	1,381 [85%]	1,275 [84%]	106 [90%]
Yes	252 [15%]	240 [16%]	12 [10%]
Housing instability in 2025			
No	1,510 [92%]	1,405 [93%]	105 [89%]
Yes	123 [8%]	110 [7%]	13 [11%]

[a] Presented are unweighted frequencies and weighted percentages.

[b] Analytic sample comprises individuals who responded to waves 4, 5, and 6, had no missing data for the primary exposure and outcome of interest, and no missing covariate data.

[c] Excluded individuals were those who had missing covariate data but otherwise responded to surveys in waves 4, 5, and 6 and had no missing data for the primary exposure and outcome of interest.

[d] Other racialized group includes those who identify as Asian, American Indian or Alaska Native, Native Hawaiian or Other Pacific Islander, multiracial, or other. Categories were collapsed due to small numbers of observations.

[e] MSA = Metropolitan statistical area

[f] Other housing tenure refers to those who reported occupying their home without payment of cash rent.

© 2026 Moon KJ et al. *JAMA Network Open*.

eTable 2. Full Regression Output From Main Model Specification

	AME [a]	Lower 95% CI [b]	Upper 95% CI [b]
Sex			
Male			0 [reference]
Female	-3.3	-5.7	-0.8
Age			
18–34 years			0 [reference]
35–44 years	0.6	-3.2	4.4
45–54 years	5.1	-3.2	13.5
55–64 years	-11.8	-22.7	-1.0
≥ 65 years	-4.8	-14.9	5.3
Racialized group			
Black	6.6	3.9	9.2
Latine	-6.7	-9.9	-3.6
Non-Hispanic white			0 [reference]
Other [c]	-0.3	-15.2	14.7
Marital status			
Divorced, separated, or widowed	2.0	-2.6	6.5
Married			0 [reference]
Never married	-1.4	-4.3	1.6
Children in the home			
No			0 [reference]
Yes, ≥ 1	-0.7	-7.8	6.4
Household size			
1 person			0 [reference]
2 persons	-0.9	-11.5	9.6
3–4 persons	4.4	-6.3	15.1
≥ 5 persons	0.8	-14.4	16.0
Educational attainment			
Less than high school			0 [reference]
High school	9.3	5.5	13.1
Some college	7.2	-2.7	17.1
Bachelor’s degree	11.7	9.6	13.7
Postgraduate degree	15.5	8.3	22.6
Employment status			
Employed			0 [reference]
Unemployed	1.7	-8.0	11.5
Retired	-13.2	-23.5	-2.9
Other	13.3	2.6	23.9
Health insurance			

© 2026 Moon KJ et al. *JAMA Network Open*.

Commercial			0 [reference]
Medicaid	14.1	9.3	18.9
Medicare	-0.2	-7.6	7.2
Uninsured	7.5	1.8	13.2
Other	15.2	1.2	29.3
Annual household income			
< $30,000			0 [reference]
$30,000–59,999	0.5	-6.6	7.5
$60,000–99,999	-0.8	-13.8	12.3
≥ $100,000	0.0	-36.0	36.0
Household savings			
< $5,000			0 [reference]
$5,000–19,999	-2.3	-7.5	3.0
$20,000–54,999	-12.4	-14.7	-10.2
$55,000–99,999	-7.6	-11.2	-4.1
≥ $100,000	-16.3	-23.1	-9.5
Census region			
Northeast			0 [reference]
Midwest	-1.3	-12.3	9.7
South	-5.8	-9.0	-2.6
West	4.7	-6.3	15.6
MSA designation [d]			
Nonmetropolitan area			0 [reference]
Metropolitan area	1.6	-6.4	9.7
Housing tenure			
Own			0 [reference]
Rent or other [e]	12.2	6.3	18.0
Prior medical debt			
No			0 [reference]
Yes	10.1	7.3	12.9
Prior housing instability			
No			0 [reference]
Yes	14.3	9.0	19.6

[a] AME = Average marginal effect
[b] CI = Confidence interval
[c] Other housing tenure refers to those who reported occupying their home without payment of cash rent
[d] MSA = Metropolitan statistical area
[e] Other housing tenure refers to those who reported occupying their home without payment of cash rent.

© 2026 Moon KJ et al. *JAMA Network Open*.

eTable 3. Full Regression Output From Model With 90% Winsorization [a]

	AME [b]	Lower 95% CI [c]	Upper 95% CI [c]
Sex			
Male			0 [reference]
Female	-4.0	-6.5	-1.4
Age			
18–34 years			0 [reference]
35–44 years	-1.7	-7.7	4.3
45–54 years	-0.1	-9.3	9.0
55–64 years	-10.9	-22.8	1.0
≥ 65 years	-4.6	-13.4	4.2
Racialized group			
Black	4.8	1.7	7.9
Latine	-2.5	-4.1	-0.9
Non-Hispanic white			0 [reference]
Other [d]	1.2	-16.1	18.5
Marital status			
Divorced, separated, or widowed	5.3	2.1	8.4
Married			0 [reference]
Never married	-1.2	-5.4	3.0
Children in the home			
No			0 [reference]
Yes, ≥ 1	2.0	-3.7	7.8
Household size			
1 person			0 [reference]
2 persons	-1.5	-11.9	8.9
3–4 persons	1.3	-8.8	11.4
≥ 5 persons	-0.1	-12.9	12.7
Educational attainment			
Less than high school			0 [reference]
High school	12.5	7.6	17.4
Some college	11.5	2.9	20.1
Bachelor’s degree	14.3	12.8	15.9
Postgraduate degree	18.3	13.3	23.4
Employment status			
Employed			0 [reference]
Unemployed	3.7	-4.3	11.8
Retired	-13.8	-23.8	-3.8
Other	10.6	1.6	19.6
Health insurance			
Commercial			0 [reference]

© 2026 Moon KJ et al. *JAMA Network Open*.

Medicaid	11.9	6.8	17.0
Medicare	-3.4	-9.7	2.9
Uninsured	4.6	0.9	8.3
Other	8.0	-1.6	17.6
Annual household income			
< $30,000			0 [reference]
$30,000–59,999	1.6	-6.9	10.0
$60,000–99,999	1.2	-12.0	14.5
≥ $100,000	1.6	-31.8	35.1
Household savings			
< $5,000			0 [reference]
$5,000–19,999	-2.7	-5.5	0.2
$20,000–54,999	-12.8	-16.0	-9.6
$55,000–99,999	-7.8	-12.4	-3.1
≥ $100,000	-17.8	-21.7	-13.9
Census region			
Northeast			0 [reference]
Midwest	1.4	-6.9	9.8
South	-0.4	-2.9	2.0
West	7.5	-1.8	16.8
MSA designation [e]			
Nonmetropolitan area			0 [reference]
Metropolitan area	0.7	-10.8	12.1
Housing tenure			
Own			0 [reference]
Rent or other [f]	11.5	5.8	17.2
Prior medical debt			
No			0 [reference]
Yes	10.1	6.3	13.9
Prior housing instability			
No			0 [reference]
Yes	14.5	9.1	19.9

[a] Winsorization procedure replaces propensity score weights below the fifth percentile or above the ninety-fifth percentile with the value of the fifth or ninety-fifth percentile, respectively, to assess sensitivity to outlier weights.
[b] AME = average marginal effect
[c] CI = confidence interval
[d] Other racialized group includes those who identify as Asian, American Indian or Alaska Native, Native Hawaiian or Other Pacific Islander, multiracial, or other.
[e] MSA = metropolitan statistical area
[f] Other housing tenure refers to those who reported occupying their home without payment of cash rent.

© 2026 Moon KJ et al. *JAMA Network Open*.

eTable 4. Association of Medical Debt With Subsequent Housing Instability, Using Propensity Score- and Survey-Weighted Logistic Regression Models

Propensity score approach	AME [95% CI] [a]
Weighting by the odds with generalized boosted models [b]	7.0 [5.2, 8.8]
Weighting by the odds with logistic regression	5.4 [3.1, 7.8]
IPTW with generalized boosted models [c]	6.5 [3.1, 9.9]
IPTW with logistic regression [c]	7.1 [5.1, 9.1]
1:1 nearest neighbor matching without replacement	6.8 [4.8, 8.9]

[a] AME = average marginal effect. CI = confidence interval
[b] Primary model presented in the main text
[c] IPTW = inverse probability of treatment weighting

© 2026 Moon KJ et al. *JAMA Network Open*.

eTable 5. Full Regression Output From Model Using Propensity Score Weighting by the Odds With Logistic Regression to Estimate ATT [a]

	AME [b]	Lower 95% CI [c]	Upper 95% CI [c]
Sex			
Male			0 [reference]
Female	-3.7	-5.5	-1.9
Age			
18–34 years			0 [reference]
35–44 years	0.8	-5.6	7.1
45–54 years	6.7	-3.3	16.7
55–64 years	-14.3	-29.2	0.6
≥ 65 years	-6.4	-14.8	2.0
Racialized group			
Black	8.0	4.3	11.6
Latine	-8.4	-9.6	-7.1
Non-Hispanic white			0 [reference]
Other [d]	0.4	-15.0	15.7
Marital status			
Divorced, separated, or widowed	1.1	-2.3	4.5
Married			0 [reference]
Never married	-3.6	-5.9	-1.2
Children in the home			
No			0 [reference]
Yes, ≥ 1	-3.5	-9.5	2.5
Household size			
1 person			0 [reference]
2 persons	2.4	-9.0	13.8
3–4 persons	9.0	-4.7	22.7
≥ 5 persons	3.3	-13.7	20.3
Educational attainment			
Less than high school			0 [reference]
High school	2.6	-2.3	7.5
Some college	0.2	-9.0	9.3
Bachelor’s degree	6.1	3.8	8.3
Postgraduate degree	7.7	0.8	14.7
Employment status			
Employed			0 [reference]
Unemployed	-0.3	-9.4	8.9
Retired	-16.4	-24.2	-8.5
Other	14.7	1.5	27.9

© 2026 Moon KJ et al. *JAMA Network Open*.

Health insurance			
Commercial			0 [reference]
Medicaid	14.8	9.4	20.2
Medicare	2.9	-3.1	8.9
Uninsured	9.4	4.1	14.6
Other	17.7	0.8	34.5
Annual household income			
< $30,000			0 [reference]
$30,000–59,999	1.9	-4.3	8.2
$60,000–99,999	-0.1	-12.7	12.4
≥ $100,000	0.6	-36.8	38.0
Household savings			
< $5,000			0 [reference]
$5,000–19,999	-1.5	-7.2	4.1
$20,000–54,999	-14.4	-16.5	-12.4
$55,000–99,999	-8.4	-12.4	-4.3
≥ $100,000	-18.0	-27.6	-8.4
Census region			
Northeast			0 [reference]
Midwest	-4.6	-17.6	8.5
South	-9.8	-16.1	-3.6
West	1.3	-10.3	13.0
MSA designation [e]			
Nonmetropolitan area			0 [reference]
Metropolitan area	2.2	-4.4	8.8
Housing tenure			
Own			0 [reference]
Rent or other [f]	13.6	7.1	20.1
Prior medical debt			
No			0 [reference]
Yes	10.2	6.2	14.1
Prior housing instability			
No			0 [reference]
Yes	15.7	11.0	20.4

[a] ATT = average treatment effect on the treated
[b] AME = average marginal effect
[c] CI = confidence interval
[d] Other racialized group includes those who identify as Asian, American Indian or Alaska Native, Native Hawaiian or Other Pacific Islander, multiracial, or other.
[e] MSA = metropolitan statistical area
[f] Other housing tenure refers to those who reported occupying their home without payment of cash rent.

© 2026 Moon KJ et al. *JAMA Network Open*.

eTable 6. Full Regression Output From Model Using Inverse Probability of Treatment Weighting With Generalized Boosted Models to Estimate ATE [a]

	AME [b]	Lower 95% CI [c]	Upper 95% CI [c]
Sex			
Male			0 [reference]
Female	-1.8	-3.6	0.1
Age			
18–34 years			0 [reference]
35–44 years	0.0	-3.1	3.1
45–54 years	-1.3	-3.0	0.3
55–64 years	-3.9	-6.8	-1.1
≥ 65 years	-2.9	-8.2	2.4
Racialized group			
Black	6.7	2.9	10.4
Latine	0.2	-0.6	1.1
Non-Hispanic white			0 [reference]
Other [d]	-1.4	-10.4	7.6
Marital status			
Divorced, separated, or widowed	3.1	0.4	5.9
Married			0 [reference]
Never married	2.1	0.0	4.1
Children in the home			
No			0 [reference]
Yes, ≥ 1	-1.7	-3.1	-0.2
Household size			
1 person			0 [reference]
2 persons	4.2	1.8	6.6
3–4 persons	3.3	1.8	4.8
≥ 5 persons	3.6	-2.2	9.3
Educational attainment			
Less than high school			0 [reference]
High school	5.5	4.0	7.0
Some college	5.3	3.8	6.7
Bachelor’s degree	2.7	1.3	4.1
Postgraduate degree	1.6	-5.9	9.0
Employment status			
Employed			0 [reference]
Unemployed	4.6	0.2	8.9
Retired	-9.0	-12.4	-5.6
Other	3.6	0.6	6.7
Health insurance			

© 2026 Moon KJ et al. *JAMA Network Open*.

Commercial			0 [reference]
Medicaid	8.2	4.8	11.6
Medicare	0.5	-2.9	3.8
Uninsured	3.9	0.1	7.6
Other	4.1	1.6	6.5
Annual household income			
< $30,000			0 [reference]
$30,000–59,999	-1.6	-6.7	3.5
$60,000–99,999	2.8	-7.5	13.0
≥ $100,000	5.8	-13.5	25.1
Household savings			
< $5,000			0 [reference]
$5,000–19,999	-3.8	-10.1	2.5
$20,000–54,999	-8.4	-11.8	-5.0
$55,000–99,999	0.1	-5.5	5.7
≥ $100,000	-5.5	-8.5	-2.5
Census region			
Northeast			0 [reference]
Midwest	-0.7	-8.6	7.2
South	-1.9	-4.5	0.8
West	3.2	-1.5	7.8
MSA designation [e]			
Nonmetropolitan area			0 [reference]
Metropolitan area	-3.6	-6.1	-1.0
Housing tenure			
Own			0 [reference]
Rent or other [f]	7.3	5.6	9.0
Prior medical debt			
No			0 [reference]
Yes	6.1	3.4	8.8
Prior housing instability			
No			0 [reference]
Yes	13.1	9.2	16.9

[a] ATE = average treatment effect
[b] AME = average marginal effect
[c] CI = confidence interval
[d] Other racialized group includes those who identify as Asian, American Indian or Alaska Native, Native Hawaiian or Other Pacific Islander, multiracial, or other.
[e] MSA = metropolitan statistical area
[f] Other housing tenure refers to those who reported occupying their home without payment of cash rent.

© 2026 Moon KJ et al. *JAMA Network Open*.

eTable 7. Full Regression Output From Model Using Inverse Probability of Treatment Weighting With Logistic Regression to Estimate ATE [a]

	AME [b]	Lower 95% CI [c]	Upper 95% CI [c]
Sex			
Male			0 [reference]
Female	-1.6	-3.9	0.7
Age			
18–34 years			0 [reference]
35–44 years	-2.1	-4.2	0.1
45–54 years	-2.7	-5.0	-0.4
55–64 years	-7.5	-8.9	-6.0
≥ 65 years	-6.6	-14.5	1.3
Racialized group			
Black	8.0	2.7	13.4
Latine	-1.5	-4.5	1.5
Non-Hispanic white			0 [reference]
Other [d]	-3.9	-16.9	9.2
Marital status			
Divorced, separated, or widowed	1.9	-1.4	5.3
Married			0 [reference]
Never married	0.6	-1.5	2.7
Children in the home			
No			0 [reference]
Yes, ≥ 1	-3.4	-5.5	-1.3
Household size			
1 person			0 [reference]
2 persons	6.1	3.4	8.7
3–4 persons	7.2	5.7	8.7
≥ 5 persons	5.4	-2.8	13.7
Educational attainment			
Less than high school			0 [reference]
High school	1.2	-1.8	4.3
Some college	1.2	-2.6	5.0
Bachelor's degree	-1.3	-3.3	0.7
Postgraduate degree	-1.5	-10.7	7.7
Employment status			
Employed			0 [reference]
Unemployed	8.5	3.2	13.7
Retired	-10.3	-13.4	-7.2
Other	6.5	3.3	9.8
Health insurance			

© 2026 Moon KJ et al. *JAMA Network Open*.

Commercial			0 [reference]
Medicaid	7.3	4.4	10.3
Medicare	2.9	0.1	5.8
Uninsured	0.3	-3.1	3.7
Other	3.6	1.3	5.8
Annual household income			
< $30,000			0 [reference]
$30,000–59,999	0.1	-5.3	5.6
$60,000–99,999	3.6	-7.5	14.6
≥ $100,000	8.7	-11.2	28.6
Household savings			
< $5,000			0 [reference]
$5,000–19,999	-5.0	-10.8	0.7
$20,000–54,999	-13.7	-17.1	-10.2
$55,000–99,999	6.9	0.9	13.0
≥ $100,000	-7.0	-8.8	-5.2
Census region			
Northeast			0 [reference]
Midwest	-2.3	-11.4	6.8
South	-5.3	-8.8	-1.9
West	4.9	-1.8	11.6
MSA designation [e]			
Nonmetropolitan area			0 [reference]
Metropolitan area	-3.4	-6.1	-0.7
Housing tenure			
Own			0 [reference]
Rent or other [f]	7.7	4.4	11.1
Prior medical debt			
No			0 [reference]
Yes	7.0	2.7	11.4
Prior housing instability			
No			0 [reference]
Yes	15.7	13.2	18.3

[a] ATE = average treatment effect
[b] AME = average marginal effect
[c] CI = confidence interval
[d] Other racialized group includes those who identify as Asian, American Indian or Alaska Native, Native Hawaiian or Other Pacific Islander, multiracial, or other.
[e] MSA = metropolitan statistical area
[f] Other housing tenure refers to those who reported occupying their home without payment of cash rent.

© 2026 Moon KJ et al. *JAMA Network Open*.

eTable 8. Full Regression Output From Model Using 1:1 Nearest Neighbor Propensity Score Matching Without Replacement

	AME [a]	Lower 95% CI [b]	Upper 95% CI [b]
Sex			
Male			0 [reference]
Female	-4.7	-5.7	0.0
Age			
18–34 years			0 [reference]
35–44 years	-1.7	-3.2	4.0
45–54 years	0.0	-3.2	5.3
55–64 years	-8.4	-22.7	4.1
≥ 65 years	1.6	-14.9	18.0
Racialized group			
Black	8.3	3.9	15.6
Latine	-0.9	-9.9	1.4
Non-Hispanic white			0 [reference]
Other [c]	1.5	-15.2	16.1
Marital status			
Divorced, separated, or widowed	6.8	-2.6	10.3
Married			0 [reference]
Never married	0.9	-4.3	4.2
Children in the home			
No			0 [reference]
Yes, ≥ 1	4.0	-7.8	9.9
Household size			
1 person			0 [reference]
2 persons	4.0	-11.5	12.2
3–4 persons	2.5	-6.3	11.9
≥ 5 persons	1.4	-14.4	12.3
Educational attainment			
Less than high school			0 [reference]
High school	10.4	5.5	16.4
Some college	5.4	-2.7	19.1
Bachelor's degree	7.9	9.6	13.6
Postgraduate degree	12.9	8.3	17.9
Employment status			
Employed			0 [reference]
Unemployed	7.5	-8.0	17.7
Retired	-14.1	-23.5	-7.7
Other	15.2	2.6	29.4

© 2026 Moon KJ et al. *JAMA Network Open*.

Health insurance			
Commercial			0 [reference]
Medicaid	10.4	9.3	16.0
Medicare	0.6	-7.6	6.5
Uninsured	12.5	1.8	16.5
Other	12.9	1.2	29.4
Annual household income			
< $30,000			0 [reference]
$30,000–59,999	0.5	-6.6	7.5
$60,000–99,999	4.6	-13.8	16.6
≥ $100,000	2.4	-36.0	33.2
Household savings			
< $5,000			0 [reference]
$5,000–19,999	2.9	-7.5	5.0
$20,000–54,999	-11.5	-14.7	-8.7
$55,000–99,999	-0.1	-11.2	3.0
≥ $100,000	-14.7	-23.1	-7.4
Census region			
Northeast			0 [reference]
Midwest	4.8	-12.3	12.1
South	3.9	-9.0	6.6
West	8.7	-6.3	17.2
MSA designation [d]			
Nonmetropolitan area			0 [reference]
Metropolitan area	0.4	-6.4	10.6
Housing tenure			
Own			0 [reference]
Rent or other [e]	11.6	6.3	18.4
Prior medical debt			
No			0 [reference]
Yes	11.0	7.3	14.5
Prior housing instability			
No			0 [reference]
Yes	15.3	9.0	21.4

[a] AME = Average marginal effect
[b] CI = Confidence interval
[c] Other racialized group includes those who identify as Asian, American Indian or Alaska Native, Native Hawaiian or Other Pacific Islander, multiracial, or other.
[d] MSA = Metropolitan statistical area
[e] Other housing tenure refers to those who reported occupying their home without payment of cash rent.

© 2026 Moon KJ et al. *JAMA Network Open*.

eTable 9. Matched Cohort of Individuals With and Without Medical Debt, Using 1:1 Nearest Neighbor Propensity Score Matching [a]

	Overall (n = 240)	No medical debt in 2024 (n = 240)	Medical debt in 2024 (n = 240)
Sex			
Male	211 [43.3%]	111 [41.9%]	100 [44.8%]
Female	269 [56.7%]	129 [58.1%]	140 [55.2%]
Age			
18–34 years	92 [25.0%]	49 [25.0%]	43 [25.0%]
35–44 years	110 [19.0%]	55 [17.9%]	55 [20.0%]
45–54 years	128 [26.9%]	63 [27.2%]	65 [26.6%]
55–64 years	95 [17.0%]	43 [16.8%]	52 [17.3%]
≥ 65 years	55 [12.1%]	30 [13.1%]	25 [11.1%]
Racialized group			
Black	320 [62.4%]	155 [61.3%]	165 [63.4%]
Latine	48 [11.5%]	22 [11.8%]	26 [11.2%]
Non-Hispanic white	87 [19.8%]	49 [20.5%]	38 [19.0%]
Other [b]	25 [6.3%]	14 [6.3%]	11 [6.3%]
Marital status			
Married	249 [48.2%]	123 [47.0%]	126 [49.3%]
Never married	118 [28.9%]	64 [29.7%]	54 [28.2%]
Divorced, separated, or widowed	113 [22.9%]	53 [23.3%]	60 [22.5%]
Children in the home			
No	286 [59.3%]	137 [58.3%]	149 [60.3%]
Yes, ≥ 1	194 [40.7%]	103 [41.7%]	91 [39.7%]
Household size			
1 person	60 [11.7%]	33 [11.9%]	27 [11.6%]
2 persons	143 [28.9%]	69 [28.0%]	74 [29.7%]
3–4 persons	190 [40.3%]	97 [42.0%]	93 [38.5%]
≥ 5 persons	87 [19.1%]	41 [18.1%]	46 [20.2%]
Educational attainment			
Less than high school	20 [7.1%]	9 [6.6%]	11 [7.6%]
High school	98 [36.4%]	49 [36.3%]	49 [36.5%]
Some college	217 [30.1%]	103 [29.2%]	114 [31.0%]
Bachelor’s degree	103 [19.3%]	58 [21.4%]	45 [17.3%]
Postgraduate degree	42 [7.1%]	21 [6.5%]	21 [7.7%]
Employment status			
Employed	316 [64.5%]	162 [65.6%]	154 [63.4%]
Unemployed	43 [10.5%]	18 [10.6%]	25 [10.4%]
Retired	48 [8.9%]	25 [8.3%]	23 [9.5%]
Other	73 [16.1%]	35 [15.5%]	38 [16.7%]

© 2026 Moon KJ et al. *JAMA Network Open*.

Health insurance			
Commercial	270 [52.2%]	139 [53.0%]	131 [51.4%]
Medicare	108 [23.9%]	54 [23.6%]	54 [24.1%]
Medicaid	51 [13.3%]	25 [13.2%]	26 [13.4%]
Uninsured	35 [7.8%]	16 [7.6%]	19 [8.0%]
Other	16 [2.8%]	6 [2.5%]	10 [3.2%]
Annual household income			
< $30,000	96 [22.4%]	49 [23.4%]	47 [21.4%]
$30,000–59,999	178 [37.9%]	85 [35.9%]	93 [39.9%]
$60,000–99,999	142 [27.9%]	74 [29.7%]	68 [26.1%]
≥ $100,000	64 [11.8%]	32 [11.0%]	32 [12.6%]
Household savings			
< $5,000	236 [50.5%]	110 [49.0%]	126 [51.9%]
$5,000–19,999	104 [22.8%]	57 [25.6%]	47 [20.2%]
$20,000–54,999	69 [14.6%]	41 [15.2%]	28 [14.1%]
$55,000–99,999	23 [3.8%]	11 [3.5%]	12 [4.0%]
≥ $100,000	48 [8.3%]	21 [6.8%]	27 [9.8%]
Census region			
Northeast	76 [19.7%]	38 [20.7%]	38 [18.8%]
Midwest	136 [20.4%]	68 [20.9%]	68 [20.0%]
South	169 [41.5%]	78 [38.8%]	91 [44.1%]
West	99 [18.4%]	56 [19.6%]	43 [17.1%]
MSA designation [c]			
Nonmetropolitan area	92 [18.6%]	45 [17.5%]	47 [19.7%]
Metropolitan area	388 [81.4%]	195 [82.5%]	193 [80.3%]
Housing tenure			
Own	268 [56.4%]	132 [55.9%]	136 [56.9%]
Rent or other [d]	212 [43.6%]	108 [44.1%]	104 [43.1%]
Prior medical debt			
No	245 [50.1%]	149 [62.6%]	96 [37.8%]
Yes	235 [49.9%]	91 [37.4%]	144 [62.2%]
Prior housing instability			
No	379 [77.4%]	205 [83.1%]	174 [71.7%]
Yes	101 [22.6%]	35 [16.9%]	66 [28.3%]
Housing instability in 2025			
No	395 [81.3%]	215 [86.2%]	180 [76.5%]
Yes	85 [18.7%]	25 [13.8%]	60 [23.5%]

[a] Presented are unweighted frequencies and weighted percentages.
[b] Other racialized group includes those who identify as Asian, American Indian or Alaska Native, Native Hawaiian or Other Pacific Islander, multiracial, or other.
[c] MSA = Metropolitan statistical area
[d] Other housing tenure refers to those who reported occupying their home without payment of cash rent.

© 2026 Moon KJ et al. *JAMA Network Open*.

eTable 10. Average Marginal Effects From Covariates in the Falsification Test, Using ChatGPT Use in 2025 as the Placebo Outcome

	AME [a]	Lower 95% CI [b]	Upper 95% CI [b]
Sex			
Male			0 [reference]
Female	-1.6	-9.4	6.2
Age			
18–34 years			0 [reference]
35–44 years	-11.0	-18.6	-3.5
45–54 years	-18.7	-25.0	-12.3
55–64 years	-35.9	-42.1	-29.8
≥ 65 years	-23.9	-43.7	-4.1
Racialized group			
Black	-15.5	-30.3	-0.6
Latine	-3.5	-10.2	3.2
Non-Hispanic white			0 [reference]
Other [c]	-2.4	-17.6	12.8
Marital status			
Divorced, separated, or widowed	-1.3	-4.3	1.7
Married			0 [reference]
Never married	-1.0	-5.5	3.4
Children in the home			
No			0 [reference]
Yes, ≥ 1	-3.3	-16.8	10.2
Household size			
1 person			0 [reference]
2 persons	-3.2	-7.0	0.6
3–4 persons	-12.3	-23.9	-0.7
≥ 5 persons	-10.7	-26.0	4.5
Educational attainment			
Less than high school			0 [reference]
High school	-17.5	-29.4	-5.7
Some college	9.6	4.8	14.3
Bachelor's degree	18.4	7.3	29.6
Postgraduate degree	19.3	11.9	26.6
Employment status			
Employed			0 [reference]
Unemployed	-1.3	-20.2	17.6
Retired	-4.0	-16.4	8.3
Other	8.2	3.2	13.2

© 2026 Moon KJ et al. *JAMA Network Open*.

Health insurance			
Commercial			0 [reference]
Medicaid	6.3	1.2	11.4
Medicare	-6.9	-14.9	1.1
Uninsured	-8.9	-22.0	4.2
Other	-20.7	-33.7	-7.7
Annual household income			
< $30,000			0 [reference]
$30,000–59,999	21.4	17.7	25.1
$60,000–99,999	22.2	11.7	32.7
≥ $100,000	27.2	17.0	37.4
Household savings			
< $5,000			0 [reference]
$5,000–19,999	8.9	-1.6	19.4
$20,000–54,999	-1.1	-11.1	8.9
$55,000–99,999	2.6	-10.5	15.6
≥ $100,000	7.1	-3.8	18.0
Census region			
Northeast			0 [reference]
Midwest	-7.4	-20.7	5.9
South	4.2	-14.1	22.6
West	-0.5	-15.4	14.4
MSA designation [d]			
Nonmetropolitan area			0 [reference]
Metropolitan area	10.0	-1.8	21.8
Housing tenure			
Own			0 [reference]
Rent or other [e]	2.9	-1.5	7.4
Prior medical debt			
No			0 [reference]
Yes	5.4	0.6	10.2
Prior housing instability			
No			0 [reference]
Yes	1.2	-14.8	17.2

[a] AME = Average marginal effect
[b] CI = Confidence interval
[c] Other racialized group includes those who identify as Asian, American Indian or Alaska Native, Native Hawaiian or Other Pacific Islander, multiracial, or other.
[d] MSA = Metropolitan statistical area
[e] Other housing tenure refers to those who reported occupying their home without payment of cash rent.

© 2026 Moon KJ et al. *JAMA Network Open*.
