# Supplementary material for: Housing Instability Following Medical Debt Exposure Among US Adults, 2023 to 2025
Source: JAMA Netw Open. 2026 Jan 12;9(1):e2553617. doi: 10.1001/jamanetworkopen.2025.53617 (PMC12797096; doi:10.1001/jamanetworkopen.2025.53617)
Supplement: Supplement 2. — Data Sharing Statement [file jamanetwopen-e2553617-s002.pdf]

Data Sharing Statement

Moon. Housing Instability Following Medical Debt Exposure Among US Adults, 2023 to 2025. *JAMA Netw Open*. Published January 12, 2026. doi:10.1001/jamanetworkopen.2025.53617

Data
Data available: Yes
Data types: Deidentified participant data, Data dictionary
How to access data: Data are available upon reasonable request for research purposes sent to CKE: cettman1@jhu.edu
When available: With publication

Supporting Documents
Document types: None

Additional Information
Who can access the data: Researchers whose proposed use of the data has been approved.
Types of analyses: For specific research analyses approved by study team.
Mechanisms of data availability: After approval of a proposal and signed data access agreement.
